# Genotypic and Phenotypic Changes in *Candida albicans* as a Result of Cold Plasma Treatment

**DOI:** 10.3390/ijms21218100

**Published:** 2020-10-30

**Authors:** Ewa Tyczkowska-Sieroń, Tadeusz Kałużewski, Magdalena Grabiec, Bogdan Kałużewski, Jacek Tyczkowski

**Affiliations:** 1 Department of Biology and Parasitology, Medical University of Lodz, Żeligowski Str. 7/9, 90-752 Lodz, Poland; ewa.tyczkowska-sieron@umed.lodz.pl; 2 Laboratory of Medical Genetics of the “Genos” Partnership - R&D Division, Inowrocławska Str. 9/132, 91-033 Lodz, Poland; t.kaluzewski@genos.com.pl (T.K.); magrabiec@gmail.com (M.G.); b.kaluzewski42@gmail.com (B.K.); 3 Department of Molecular Engineering, Faculty of Process and Environmental Engineering, Lodz University of Technology, Wólczańska Str. 213, 90-924 Lodz, Poland

**Keywords:** *Candida albicans*, cold plasma treatment, genome, hydrolytic enzyme activity, carbon assimilation, drug susceptibility

## Abstract

We treated *Candida albicans* cells with a sublethal dose of nonequilibrium (cold) atmospheric-pressure He plasma and studied alterations in the genome of this fungus as well as changes in the phenotypic traits, such as assimilation of carbon from carbohydrates, hydrolytic enzyme activity, and drug susceptibility. There is a general problem if we use cold plasma to kill microorganism cells and some of them survive the process—whether the genotypic and phenotypic features of the cells are significantly altered in this case, and, if so, whether these changes are environmentally hazardous. Our molecular genetic studies have identified six single nucleotide variants, six insertions, and five deletions, which are most likely significant changes after plasma treatment. It was also found that out of 19 tested hydrolytic enzymes, 10 revealed activity, of which nine temporarily decreased their activity and one (naphthol-AS-BI- phosphohydrolase) permanently increased activity as a result of the plasma treatment. In turn, carbon assimilation and drug susceptibility were not affected by plasma. Based on the performed studies, it can be concluded that the observed changes in *C. albicans* cells that survived the plasma action are not of significant importance to the environment, especially for the drug resistance and pathogenicity of this fungus.

## 1. Introduction

The application of nonequilibrium (cold) atmospheric-pressure plasma for the inactivation of microorganisms in the areas of medicine, biotechnology, and food processing has attracted a rapidly growing interest in recent years. In particular, much attention has been paid to plasma medicine and healthcare [1,2,3,4]. Numerous attempts to use cold plasma in oncology [5], dermatology [6,7,8], and wound healing [9], as well as in disinfection and sterilization [10,11], are already quite advanced. The studies have long since gone beyond in vitro experiments and are conducted in vivo in animals as well as in humans as clinical trials [12,13,14,15,16]. A significant problem that has emerged as a result of these studies is the finding that plasma treatment does not necessarily kill all microorganisms in the area of plasma action. Some of them survive, and it is suspected that certain changes in their genome and phenotypic traits occur [17,18]. Particularly dangerous changes would be the increase in virulence and drug resistance, which would place the application of plasma technology in a disadvantageous position in medicine. Although researchers have just turned their attention to the plasma-induced sublethal effects in living organisms [19,20], also studying them on isolated DNA [21,22,23] or enzymes [24], the precise mechanism of this phenomenon, especially the types of DNA damage, is poorly characterized [25]. This problem, therefore, requires further and more advanced studies.

Following this research trend, in this paper, we present the results of studies on *Candida albicans* cells in the sublethal state, which survived plasma treatment, looking for genotypic and phenotypic changes that would be a consequence of such a treatment. *C. albicans* was chosen for two reasons. Firstly, it is a well-characterized microorganism [26], which is recognized as a model in the study of fungal pathogens [27], but most of all—secondly—it is the most important cause of fungal infections in humans [28,29], commonly known as candidiasis, which poses a serious therapeutic problem, among others, due to its constantly increasing resistance to antifungal agents [30,31,32]. It is not surprising then that new and effective strategies of fighting against candidiasis are being searched for. Considering that superficial candidiasis, mainly skin infections, are the most numerous and widespread group of all fungal infections [33,34], cold plasma, which is especially suitable for surface treatment, seems to be a very promising therapeutic method in this case. As this method is becoming increasingly popular and is beginning to be applied in practice, the determination of changes that may occur in *C. albicans* cells that survived plasma treatment is particularly justified.

Several tests with the use of cold plasma have already been carried out on *C. albicans*. Apart from determining the influence of plasma parameters and treatment time on the survival rate of the cells of this fungus using various types of plasma sources [35,36,37,38,39], a few preliminary studies have also been carried out on the changes that occur in sublethal cells. For example, Rahimi-Verki et al. [40] found a reduction in the activity of phospholipase and proteinase enzymes for plasma-treated *C. albicans* samples compared to untreated controls. A decrease in the amount of ergosterol after plasma treatment was also noticed. On the other hand, Borges et al. [41] did not observe the effect of plasma on exoenzyme (phospholipase and proteinase) production but found a promising impact of plasma treatment on morphogenesis, with an almost 40-fold reduction of the filamentation rate compared to the nonexposed group. As can be seen, just taking enzymes as an example, the response of *C. albicans* to plasma treatment is inconclusive and requires further detailed investigation.

## 2. Results and Discussion

**Figure 1:**
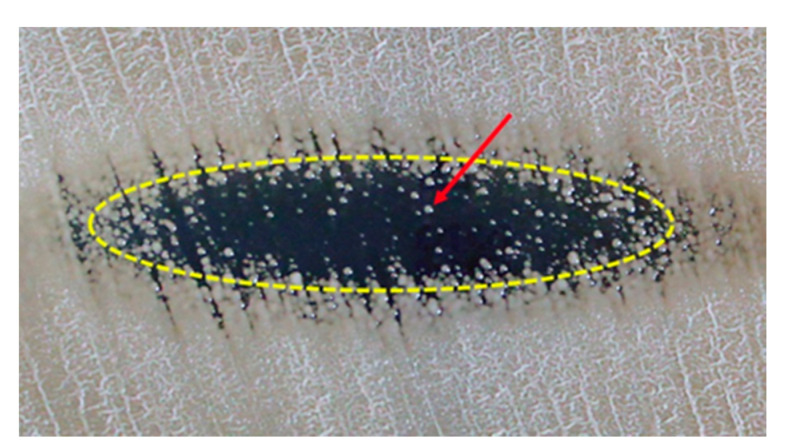
The growth inhibition zone induced by plasma that was generated by the linear microdischarge jet. The red arrow indicates one of the spot colonies, which grew after plasma treatment.

Surface plasma treatment of *C. albicans* culture induced the growth inhibition zone with an elliptical-like shape, as shown in Figure 1. The zone edge has been defined as a place where cell survival reaches an average of 10 % compared to the plasma-untreated region [39]. Due to the appropriately selected plasma parameters (see Section 3.1) and the relatively short time of plasma action (see Section 3.2), not all *C. albicans* cells in the elliptical zone were killed. Some of them survived, which were revealed, after incubation, as spot colonies inside this zone (Figure 1). These colonies were collected for further investigations.

### 2.1. Genomic Alterations

**Figure 2:**
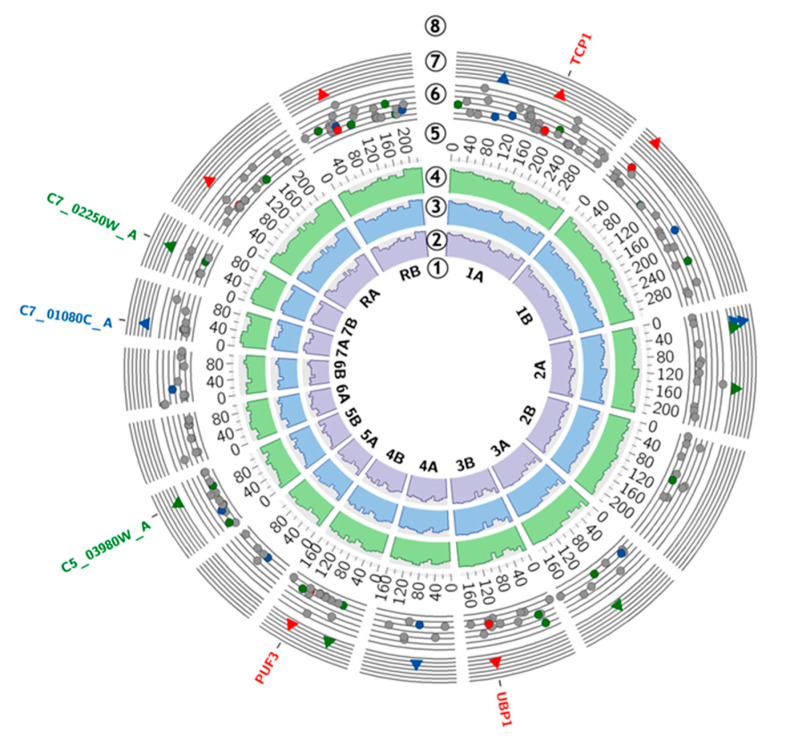
Genome diagram: (**1**) *C. albicans* SC5314 A22 ideogram; (**2**) control sample coverage; (**3**) 12_1 sample coverage; (**4**) 12_2 sample coverage; (**5**) chromosomal coordinates; (**6**) genomic alternations (grey—low confidence single nucleotide variants (SNVs), red—high confidence SNVs, blue—deletions, green—insertions); (**7**) genomic alternations statistically significant (red—high confidence SNVs, blue—deletions, green—insertions); (**8**) genes affected by high-confidence SNVs (red), deletion (blue), insertions (green).

The results of nuclear DNA sequencing are presented on a circular plot (Figure 2). The variant calling allows the identification of numerous genomic alterations in each evaluated sample. However, since no genomic changes affecting the mitochondrial genome have been recorded, mitochondrial DNA is not discussed further.

In this study, we focused on the single nucleotide variants (SNVs), insertions, and deletions, which were present in two investigated samples after 12 cycles of plasma treatment (12_1 and 12_2) and were significantly more frequent than in the control sample. This approach led to the identification of six single nucleotide variants, six insertions, and five deletions. The following genes were affected by high-confidence SNVs: TCP1 (p.Gly>Arg and p.Gly>Glu), UBP1 (p.Gln>STOP), and PUF3 (p.Gly>Gly). Short insertions were present in the sequence of genes: C5_03980W_A and C7_02250W_A. The sequence of C7_01080C_A was interfered with by deletion. All of the abovementioned mutations were not exclusive to the tested samples. They were also present in the control sample, but they were significantly less frequent, according to the VarScan software evaluation. Particular attention was paid to genes known to alter the *C. albicans* physiology—ALS1−ALS12, HWP1, EAP1, ECM33, MP65, PHR1, SAP1−SAP10, SAP30, SAP98, SAP99, SAP155, SAP190, RSR1, BIG1, LIP1−LIP10, PMT1, PMT4, BCR1, TEC1, ACE2, EFG1, ZAP1, MDR1, CDR1, CDR2, EFG1, CPH1, CPH2, ECE1, TUP1, NRG1, RFG [42], and ERG3, ERG11 [43]). There were no significant changes in the sequence of those genes. A detailed summary of the molecular findings is presented in Table 1.

The genome of *C. albicans* has a length of 28,605,418 bp (including the nuclear and mitochondrial genome) and consists of eight pairs of chromosomes [44]. A characteristic feature of this organism is the occurrence of frequent genetic alternations (translocations, insertions, and deletions on both the chromosomal and nucleotide levels), which are part of its strategy to adapt to environmental conditions [45]. The abovementioned circumstances, along with the alternative codon usage (CUG translated into serine rather than leucine), make it a challenging organism to study.

In the performed analysis, we identified several changes in the nuclear genome of the examined strain. During the analysis, we proved the presence of thousands of SNVs, insertions, and deletions in each of the samples, including the control. The genomic alternations presented in the results were selected after a detailed statistical analysis, taking into account the methodology of the experiment. However, there is no evidence that any of them appeared as a result of plasma treatment, and it is not simply a matter of coincidence. To gain a deeper understanding of possible plasma-induced genetic changes in *C. albicans*, phenotypic characteristics such as carbon assimilation, enzyme activity, and susceptibility to antifungal drugs should be investigated, and, if changes occur due to the plasma treatment, to try to link them to the genetic changes. The following part of this paper presents the results of such studies.

### 2.2. Phenotypic Changes

#### 2.2.1. Carbon Assimilation and Hydrolytic Enzyme Activity

Tests of the ability to assimilate carbohydrates as a carbon source did not show any changes in the investigated *C. albicans* strain after repeated sublethal treatments with the use of cold atmospheric plasma. The analyzes were performed for the untreated culture as well as for cultures after 1, 7, and 12 plasma treatments. Both before and after the plasma treatment, the studied fungal cells were characterized by the ability to assimilate carbon from the same 13 compounds. As an example, Table 2 shows the API 20C AUX test results for the untreated culture and after 12 cycles of plasma treatment. A plus sign indicates that *C. albicans* grows on this carbohydrate medium.

**Figure 3:**
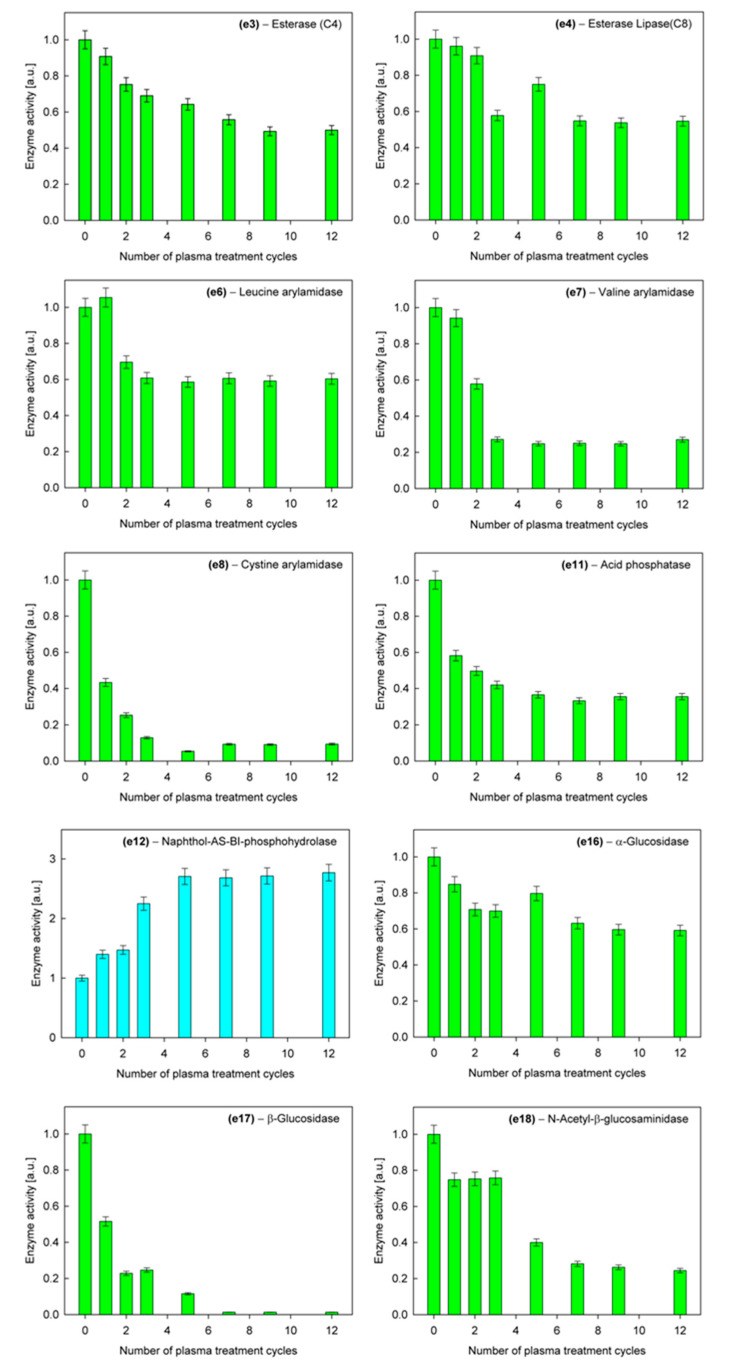
Activities of hydrolytic enzymes for C. albicans as a function of the number of plasma cycles.

The situation is different, however, in the case of the activity of hydrolytic enzymes, which is associated with the virulence of *C. albicans* [46,47,48] and therefore requires more careful analysis. Among 19 tested enzymes (Table 3), 10 of them showed activity in the investigated strain of *C. albicans*. In nine cases, the activity decreased in the course of successive cycles of plasma treatment, even falling to zero, while in one case, it increased. Relative average activities for successive plasma treatment cycles of these enzymes are shown in Figure 3.

**Figure 4:**
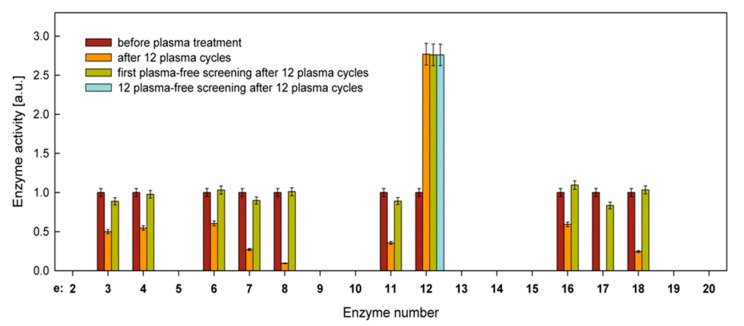
Summary of normalized activities of hydrolytic enzymes for *C. albicans*, before plasma treatment, after 12 plasma cycles, and after subsequent plasma-free screenings. All activity values for active enzymes before plasma treatment were normalized to 1.

After 12 cycles, further screening tests were performed without plasma to determine the persistence of the observed changes. As shown in Figure 4, the activity of the enzymes decreased due to the plasma action returning to the initial state, while the activity of the enzyme No. 12 (naphthol-AS-BI-phosphohydrolase), which increased, remained unchanged. The next 12 screenings did not change the activity of enzyme No. 12, indicating a permanent change.

Based on these results, we have two problems to solve. First, what is the reason for the unstable decrease in the activity of nine of the tested enzymes, which returned to their original state after the first screening without plasma treatment? Secondly, why did enzyme No. 12 permanently change its activity?

The reversible changes in enzyme activity, observed for nine enzymes, may be due to cell-to-cell transmission of information from dying cells about danger during the plasma action [49], for example, via cytoplasmic flow [50]. It can also be caused by stress induced by changes in the environment [51], for example, the byproducts of killed cells, which are constantly present during the growth of colonies after plasma treatment. The fact that virtually all cells growing in a given colony revealed reduced enzyme activity directly after plasma treatment and while being reinoculated onto a new medium, they forgot this change and returned to the state of original activity, indicates a stress effect that is not a reflection of genetic changes. To find out more precisely what could be causing the stress, the strain was inoculated on the medium that had been treated with plasma for 12 min. No changes in enzyme activity were observed in this case. This indicates that it is not the changes in the substrate due to the action of the plasma but the products derived from killed cells that are a source of stress.

On the other hand, the sustained increase in naphthol-AS-BI-phosphohydrolase activity after plasma treatment seems to have a genetic basis. Changes in the genome could be generated by both the cell-to-cell communication process during plasma operation and the stress induced by plasma products. Unfortunately, the lack of a connection in the literature between this enzyme and specific genetic sequences makes it difficult to determine the exact mechanism that caused these changes. However, it should be added that the naphthol-AS-BI-phosphohydrolase enzyme is not, so far, considered to be an important hydrolytic enzyme that affects the virulence of *C. albicans* [48,52].

#### 2.2.2. Susceptibility to Antifungal Drugs

Susceptibility studies of *C. albicans* to antifungal drugs as a function of the number of cycles of plasma treatment did not reveal any differences in comparison to the untreated culture. Table 4 shows the MIC (minimum inhibitory concentration) values determined for the tested drugs for the untreated culture and the culture after 12 plasma treatment cycles, i.e., after the highest plasma exposure we have used for cells that survived such treatment. These results were confirmed by repeating the measurement series three times. Although the dose of plasma in each cycle was large enough to kill the vast majority of cells in its area of action (Figure 1), it did not alter drug susceptibility in this small number of surviving cells. No increase in drug resistance for cells that have survived the plasma action bodes well for the future progress of using plasma techniques in the fight against superficial candidiasis.

## 3. Materials and Methods

### 3.1. Plasma Source

**Figure 5:**
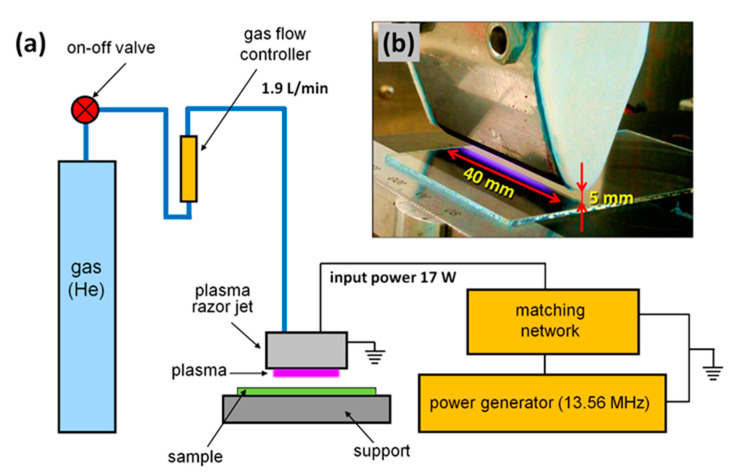
The experimental system: (**a**) schematic diagram of the setup; (**b**) photo of the plasma razor jet.

As a source of nonequilibrium (cold) atmospheric plasma for the treatment of *Candida* cells, we used a linear microdischarge jet (produced in-house), sometimes called a plasma razor jet [53]. More details on the design of the plasma jet, its principle of operation, and process characteristics can be found in [39]. In this study, plasma was generated at 13.56 MHz in helium as a working gas. The helium flow rate was 1.9 L/min, while the discharge power was set at 17 W. The plasma beam had a cylindrical shape, with a length of 40 mm and a diameter of about 1.5 mm, and was aligned parallel to the treated surface at a distance of 5 mm. The experimental system is shown in Figure 5.

### 3.2. Microbiological and Plasma Treatment Procedures

The reference strain of the American Type Culture Collection (ATCC) *Candida albicans* ATCC^®^ 10231 (National Collection of Pathogenic Fungi (NCPF), Salisbury, UK) was used in this study. A 24-h culture of the fungus for further studies was prepared by uniformly spreading 100 μL of phosphate buffer solution (PBS) containing 5 × 10^7^ CFU/mL (5 units of the McFarland scale, determined with a DEN-1B type densitometer, Biosan, Riga, Latvia) onto a Petri dish with Sabouraud dextrose agar (bioMérieux, Marcy-l’Etoile, France), after which it was exposed to the plasma treatment for 1 min and then incubated at 37 °C for 24 h. *C. albicans* cells that were not killed in the plasma-induced growth inhibition zone were the protoplasts of growing spot colonies, which were the starting material for further studies. They also served as the basis for the preparation of the next culture and its plasma treatment, exactly as it was done the first time. This procedure was repeated 12 times in each measurement series. Then, subsequent screenings without plasma treatment were performed to determine the durability of occurring changes that had taken place under the influence of the plasma. As control samples, a 24-h culture of the fungus was used.

### 3.3. DNA Isolation and Genome Bioinformatics Analysis

The molecular genetic research was based on a comparison between the control sample (“control”) and two test samples after 12 exposure periods (“12_1” and “12_2”) of the selected *Candida albicans* strain. The analysis aims to identify possible genetic changes caused by cold plasma treatment.

The DNA isolation was performed with 50 mg of 24-h cultures of the *C. albicans*, before and after plasma treatment. The biological material was triturated in liquid nitrogen, in a sterile mortar, to which the CTAB extraction buffer (OPS Diagnostics LLC, Lebanon, NJ, USA), proteinase K (Promega, Madison, WI, USA), and RNase-A (Promega, Madison, WI, USA) were added. The prepared material was transferred to sterile Eppendorf tubes and incubated in a ThermoMixer C (Eppendorf, Hamburg, Germany) at 70 °C for 30 min. The tubes were centrifuged in an Eppendorf Centrifuge. The collected 200 μL of supernatant fluid was transferred to Maxwell^®^ 16LEV Plant DNA Kit cartridges (Promega, Madison, WI, USA), and the isolation continued in a Maxwell^®^ 16 machine (Promega, Madison, WI, USA). The amount and purity of DNA were assessed using a Nanodrop ND 2000C (ThermoFisher Scientific, Waltham, MA, USA) and a Qubit fluorimeter (ThermoFisher Scientific, Waltham, MA, USA). Sequencing libraries were prepared using the TruSeq DNA PCR-free reagent set (350 bp insert; Illumina, San Diego, CA, USA), and the type of prepared libraries was Illumina-Shotgun. Next-generation sequencing was carried out on the HiSeq X Ten platform (Illumina, San Diego, CA, USA) to generate 2 × 150 bp paired-end reads, assuming a path coverage in the flow cell for each sample of 20 %.

The quality of the obtained raw reads was checked using FastQC v0.11.4 software [54]. Then, the adapter sequences and low-quality regions of raw reads were trimmed using the Trimmomatic v0.36 tool [55], with the following operating parameters: initial and final regions quality >20, average read quality >30, minimum reads length = 90. The quality of processed reads has been confirmed by rechecking the samples with the FastQC program. The obtained reads were mapped using STAR mapper v2.7.3a [56] to the current, up-to-date version of the *C. albicans* reference genome (*C. albicans* SC5314 A22) taken from the Candida Genome Database [44], implementing the default operating parameters. To identify single nucleotide variations, as well as insertions and deletions in the nuclear sequences, the data was converted to mpileup format using SAMtools v1.10 software [57]. The variant calling and comparison between the samples was performed by VarScan v2.4.4 [58], with default parameters. The effect of selected SNVs on protein products was evaluated using IGV browser v2.7.2 [59]. The obtained data were visualized in the Perl environment with the usage of Circos v.0.69.9 [60]. The coverage data for visualization was obtained by deepTools v2.0 [61].

### 3.4. Carbohydrate Assimilation Test

To evaluate the changes in the biochemical properties of the strain after plasma treatment, investigations were carried out using the API 20C AUX system (bioMérieux, Marcy-l’Etoile, France). It is a biochemical identification series based on the assessment of the ability of fungi to absorb carbon (auxanogram) from 19 compounds, i.e., D-glucose (GLU), glycerol (GLY), 2-keto-D-gluconate (2KG), L-arabinose (ARA), D-xylose (XYL), adonitol (ADO), xylitol (XLT), D-galactose (GAL), inositol (INO), D-sorbitol (SOR), methyl-αD-glucopyranoside (MDG), N-acetyl-D-glucosamine (NAG), D-cellobiose (CEL), D-lactose (LAC), D-maltose (MAL), D-sucrose (SAC), D-trehalose (TRE), D-melesitose (MLZ), and D-raffinose (RAF). The API 20C AUX strip consists of 20 cupules containing the 19 dehydrated substrates and a place for the control sample, where assimilation tests are performed. A semiliquid starvation medium is introduced into the cupules. Yeast-like fungi grow if they can use a given substrate as the only carbon source. The API strips were prepared according to the manufacturer’s instructions and incubated at 30 °C for 72 h, after which the carbohydrate assimilation patterns were read.

### 3.5. Hydrolytic Enzyme Activity 

The hydrolytic enzyme activities for the investigated fungal strain were determined using an API ZYM system (bioMérieux, Marcy-l’Etoile, France). The system enables rapid determination of the activity of 19 enzymes (5 peptidases, 3 lipases, 3 phosphatases, and 8 carbohydrases) using very small amounts of unpurified samples. The list of the enzymes is presented in Table 3. Although it is a qualitative test based on visual comparison of the colors produced by enzymatic reactions, with a printed color standard to more quantitatively determine the enzyme activities, we used spectrophotometric analysis.

Based on the manufacturer’s instructions, enzymatic activity measurements were carried out by suspending a given fungal sample in 2 mL of sterile distilled water with turbidity between 5 and 6 on the McFarland scale and then transferring 65 μL of this suspension to each cupule of the test strip. The strips were incubated for 4 h at 37 °C. After incubation, one drop of ZYM A reagent (trihydroxymethylaminomethane, 37% hydrochloric acid, lauryl sulfate, distilled water) and one drop of ZYM B reagent (fast blue 2 BB, methoxyethanol) were added to each cupule to stop the reaction. After 5 min of color development, the test strip was exposed to intense visible light for 10 s to eliminate a yellow tint due to an excess of unreacted fast blue 2 BB. Then, a 5-μL sample was taken from each cupule and placed in a microvolume Nano Stick-S for UV–vis spectrophotometry (PIKE Technologies, Fitchburg, WI, USA). The absorbance of each sample was measured using a spectrophotometer UV–VIS Jasco V-630 (ABL&E-JASCO Polska, Cracow, Poland) for the wavelength characteristic of the given enzyme reaction (Table 3) [62]. Two identical tests were performed at the same time for each sample, while each series of measurements was repeated three times. The maximum relative uncertainty in determining the activity of a given enzyme for a given sample is estimated to be ±5%.

### 3.6. Susceptibility Testing

The in-vitro activity of typical antifungal agents (voriconazole, fluconazole, amphotericin B, caspofungin, micafungin, and anidulafungin) was determined by the Etest strips (bioMérieux, Marcy- l’Etoile, France). This method consists of placing a narrow plastic strip soaked with a given agent of increasing concentration along this strip on a fungal culture. This allows us to estimate the minimum inhibitory concentration (MIC), which is a measure of drug activity. Inoculum suspensions in sterile saline (0.85 % NaCl) were prepared from primary and plasma-treated *C. albicans* cultures, with an optical density of 0.5 McFarland standard (approximately 5 × 10^6^ CFU/mL). The suspensions were inoculated directly onto plates, with RPMI-1640 agar (bioMérieux, Marcy-l’Etoile, France) as the base medium on which the Etest strips were placed, according to the manufacturer’s instructions. MIC values were read 24 h after incubation at 35 °C, accurate to scale on the Etest strips. Each series of measurements was repeated three times.

## 4. Conclusions

One of the problems associated with the development of plasma medicine is the risk that microorganisms surviving the plasma action may unfavorably change their characteristics; for example, their virulence or resistance to drugs may increase. The studies conducted on the *C. albicans* fungus have not confirmed these concerns. Although both genotypic and phenotypic changes were observed as a result of repeated plasma treatment, they did not have a significant effect on the virulence and drug susceptibility of the tested strain.

Among the observed changes, the alteration in enzyme activity is of particular interest. The results showed that 9 out of 19 tested enzymes reduced, some significantly, their activity after plasma treatment, which could be interpreted as a decrease in the virulence of this fungus. However, when such cells were inoculated onto a new medium devoid of plasma products, for example, killed cells, the activities of these enzymes returned to their original state. This most likely indicates the occurrence of a stress effect, which should be further investigated in more detail.

Another interesting result is the permanent increase in the activity of one of the enzymes (naphthol-AS-BI-phosphohydrolase) under plasma treatment. Multiple screening of cells on the medium, which had not been in contact with the plasma, did not change the elevated enzyme activity. This permanent shift in the activity must result from alterations in the genotype. Additionally, although we have identified some of the genetic alterations that were possibly plasma-induced in *C. albicans*, their link to the change in enzyme behavior, as well as explaining the genesis of this phenomenon, requires further research.

In summary, it should be noted that the performed studies, on the one hand, have shown changes in both the genotype and phenotype of *C. albicans* under the influence of plasma, which, however, are not dangerous in terms of virulence and drug resistance, and, on the other hand, have revealed some interesting effects related to the activity of hydrolytic enzymes.

## Figures and Tables

**Table 1:** Summary of statistically significant mutations detected in the tested samples.

Standard Name	Systematic Name	Localization	Mutation	Sample 12_1 *p*-Value	Sample 12_2 *p*-Value	Description [44]
Single Nucleotide Variants						
TCP1	C1_08560W_A	Chr1A:1874103	G>C (p. Gly>Arg)	0.0369	0.0095	Chaperonin- containing T-complex subunit, induced by alpha pheromone in SpiderM medium; stationary phase enriched protein
		Chr1A:1874104	G>A(p.Gly>Glu)	0.0471	0.0112	
Uncharacterized region		Chr1B:275481	T>A	0.0033	0.0104	–
UBP1	C3_05870C_A	Chr3B: 1315611	C>T (p.Gln>STOP)	0.0208	0.0119	Ortholog(s) have thiol-dependent ubiquitin-specific protease activity, role in negative regulation of protein autoubiquitination, protein deubiquitination and cytoplasm, endoplasmic reticulum localization
PUF3	C4_05370W_A	Chr4B:1172318	T>C (p.Gly>Gly)	0.0304	0.0304	RNA-binding protein involved in the regulation of mitochondrial biogenesis
Uncharacterized region		ChrRA:655094	A>G	0.0405	0.0082	–
Uncharacterized region		ChrRA:655095	C>G	0.0336	0.0031	–
**Insertions and Deletions**						
Uncharacterized region		Chr1A:837098	-AAAAGAAAAG	0.0064	0.0428	–
Uncharacterized region		Chr2A:254001	-TAGAAGAAGAAT	0.0027	0.0345	–
Uncharacterized region		Chr2A:342691	+C	0.0028	0.0257	–
Uncharacterized region		Chr2A:239531	-T	0.0105	0.0337	–
Uncharacterized region		Chr2A:1421683	+GATATTTAAGG	0.0153	0.0256	–
Uncharacterized region		Chr3A:980388	+T	0.0338	0.0291	–
Uncharacterized region		Chr4A:686524	-T	0.0373	0.0346	–
Uncharacterized region		Chr4B:456519	+TAAAT	0.0185	0.0285	–
−	C5_03980W_A	Chr5B: 878800	+CAACAACAA	0.0403	0.0364	Protein of unknown function; Spider biofilm induced
−	C7_01080C_A	Chr7A:228708	-TC	0.0182	0.0231	Major repeat sequence (MRS) on Chromosome 7; MRS units are composed of variable numbers of RPS units flanked by HOK and RB2 sequences; found on most chromosomes; may serve as recombination hot spot
–	C7_02250W_A	Chr7B:490089	+TTCCAA	0.0022	0.0208	Ortholog of *C. dubliniensis* CD36: Cd36_72050, *C. parapsilosis* CDC317: CPAR2_301140, *C. tenuis* NRRL Y-1498: CANTEDRAFT_ 135055, and *Debaryomyces hansenii* CBS767: DEHA2E07678g

**Table 2:** The ability of the tested strain to absorb carbon from 19 carbohydrates, assessed using the API 20C AUX system.

Carbohydrate	Carbohydrate Symbol	Before Plasma Treatment	After 12 × Plasma Treatment
D-glucose	GLU	+	+
glycerol	GLY	+	+
2-keto-D-gluconate	2KG	+	+
L-arabinose	ARA	−	−
D-xylose	XYL	+	+
adonitol	ADO	+	+
xylitol	XLT	+	+
D-galactose	GAL	+	+
inositol	INO	−	−
D-sorbitol	SOR	+	+
methyl-αD-glucopyranoside	MDG	+	+
N-acetyl-D-glucosamine	NAG	+	+
D-cellobiose	CEL	−	−
D-lactose	LAC	−	−
D-maltose	MAL	+	+
D-sucrose	SAC	+	+
D-trehalose	TRE	+	+
D-melesitose	MLZ	−	−
D-raffinose	RAF	−	−

**Table 3:** List of hydrolytic enzymes tested, using the API ZYM system and the characteristic wavelengths, at which the absorbance of the products of the enzymatic reactions was measured.

No.	Enzyme	Wavelength [nm]
e2	Alkaline phosphatase	537
e3	Esterase (C4)	537
e4	Esterase Lipase (C8)	537
e5	Lipase (C14)	537
e6	Leucine arylamidase	494
e7	Valine arylamidase	494
e8	Cystine arylamidase	494
e9	Trypsin	494
e10	α-Chymotrypsin	494
e11	Acid phosphatase	537
e12	Naphthol-AS-BI-phosphohydrolase	582
e13	α-Galactosidase	547
e14	β-Galactosidase	547
e15	β-Glucuronidase	582
e16	α-Glucosidase	537
e17	β-Glucosidase	537
e18	N-Acetyl-β-glucosaminidase	454
e19	α-Mannosidase	537
e20	β-Fucosidase	537

**Table 4:** Minimum inhibitory concentration (MIC) values (μg/mL) of antifungal agents for the investigated *C. albicans* strain.

Antifungal Agent	Before Plasma Treatment [MIC Value]	After 12 × Plasma Treatment [MIC Value]
Voriconazole	0.094	0.094
Fluconazole	2.0	2.0
Caspofungin	0.125	0.125
Amphotericin B	0.125	0.125
Micafungin	0.012	0.012
Anidulafungin	0.004	0.004

## Abbreviations

*C. albicans*	*Candida albicans*
CFU/mL	Colony-forming unit per milliliter
CUG	Ttrinucleotide codon composed of cytosine, uracil and guanine
L/min	Liter per minute
MHz	Megahertz (frequency unit)
MIC	Minimum inhibitory concentration
SNVs	Single nucleotide variants
W	Watt (power unit)
